# MicroRNA signatures in osteosarcoma: diagnostic insights and therapeutic prospects

**DOI:** 10.1007/s11010-024-05135-5

**Published:** 2024-10-17

**Authors:** Mritunjoy Dey, Palina Skipar, Ewa Bartnik, Jakub Piątkowski, Dorota Sulejczak, Anna M. Czarnecka

**Affiliations:** 1 Department of Soft Tissue/Bone Sarcoma and Melanoma, Maria Sklodowska-Curie National Cancer Research Institute in Warsaw, 02-781 Warsaw, Poland; 2 Faculty of Medicine, Warsaw Medical University, 02-091 Warsaw, Poland; 3 Institute of Genetics and Biotechnology, Faculty of Biology, University of Warsaw, 02-106 Warsaw, Poland; 4 Department of Experimental Pharmacology, Mossakowski Medical Research Institute, Polish Academy of Sciences, Warsaw, Poland

**Keywords:** Osteosarcoma, MicroRNA, Primary malignant bone tumor, Therapeutic targets, Drug resistance, Gene regulation

## Abstract

Osteosarcoma (OSa) is the most prevalent primary malignant bone tumor in children and adolescents, characterized by complex genetic and epigenetic alterations. Traditional treatments face significant challenges due to high rates of drug resistance and lack of targeted therapies. Recent advances in microRNA (miRNA) research have opened new avenues for understanding and treating osteosarcoma. This review explores the many critical functions of miRNAs in osteosarcoma, particularly their potential for clinical use. The review highlights two key areas where miRNAs could be beneficial. Firstly, miRNAs can act as biomarkers for diagnosing osteosarcoma and predicting patient prognosis. Secondly, specific miRNAs can regulate cellular processes like proliferation, cell death, migration, and even resistance to chemotherapy drugs in osteosarcoma. This ability to target multiple pathways within cancer cells makes miRNA-based therapies highly promising. Additionally, though the interaction between miRNAs and circular RNAs (circRNAs) falls outside the scope of the paper, it has also been discussed briefly. While miRNA-based therapies offer exciting possibilities for targeting multiple pathways in osteosarcoma, challenges remain. Efficient delivery, potential off-target effects, tumor complexity, and rigorous testing are hurdles to overcome before these therapies can reach patients. Despite these challenges, continued research and collaboration among scientists, clinicians, and regulatory bodies hold the promise of overcoming them. This collaborative effort can pave the way for the development of safe and effective miRNA-based treatments for osteosarcoma.

## Introduction

Osteosarcoma (OSa) is the most common primary bone cancer among adolescents and young adults. While surgery combined with standard chemotherapy (MAP regimen) improves outcomes for localized tumours, 40–50% of patients experience relapse, leading to poor prognosis, especially in metastatic cases [1, 2]. Additionally, the use of chemotherapy upon relapse was significantly associated with the inability to achieve complete remission [3]. The American Cancer Society reports 5-year survival-rates of 76%, 64%, and 24% for localized, regional, and distant osteosarcoma, respectively [4]. Unfortunately, no major therapeutic advancements have been made in the past 30 years, largely due to the absence of reliable outcome biomarkers to guide patient stratification for new therapies [5–7].

Preoperative chemotherapy, aimed at reducing the primary tumor volume, is a cornerstone of OSa treatment. This approach involves combinations of chemotherapeutic agents, such as cisplatin, doxorubicin, ifosfamide, and methotrexate, with the goal of maximizing tumor necrosis and preventing chemoresistance [8, 9]. Following a month of chemotherapy, surgical removal of the tumor is performed, followed by postoperative chemotherapy [10]. However, the evaluation of pathologic necrosis after neoadjuvant chemotherapy, while somewhat indicative of prognosis, has significant limitations. It is subjective, requires expert evaluation, and does not reliably predict outcomes for patients with suboptimal responses. The EURAMOS trial, which used necrosis for patient stratification in adjuvant therapy, failed to demonstrate a survival benefit, underscoring the need for new therapies and better prognostic markers [11].

In this context, microRNAs (miRs) have gained attention as potential biomarkers and therapeutics. These short, non-coding, endogenous, and highly conserved RNA molecules are crucial in regulating post-transcriptional gene expression. They exhibit distinct expression patterns during neoplasia and are established regulators of carcinogenesis, typically modulating gene expression by binding to the untranslated regions of target messenger RNAs (mRNAs), resulting in translational repression or mRNA destabilization [12]. Beyond these classical roles, miRNAs can exert atypical effects, such as activating gene expression by binding to specific promoters or interacting with transcriptional machinery. They also regulate long non-coding RNAs (lncRNAs) and circular RNAs (circRNAs), which act as competitive inhibitors or sponges, influencing miRNA availability and function [13].

**Fig. 1:**
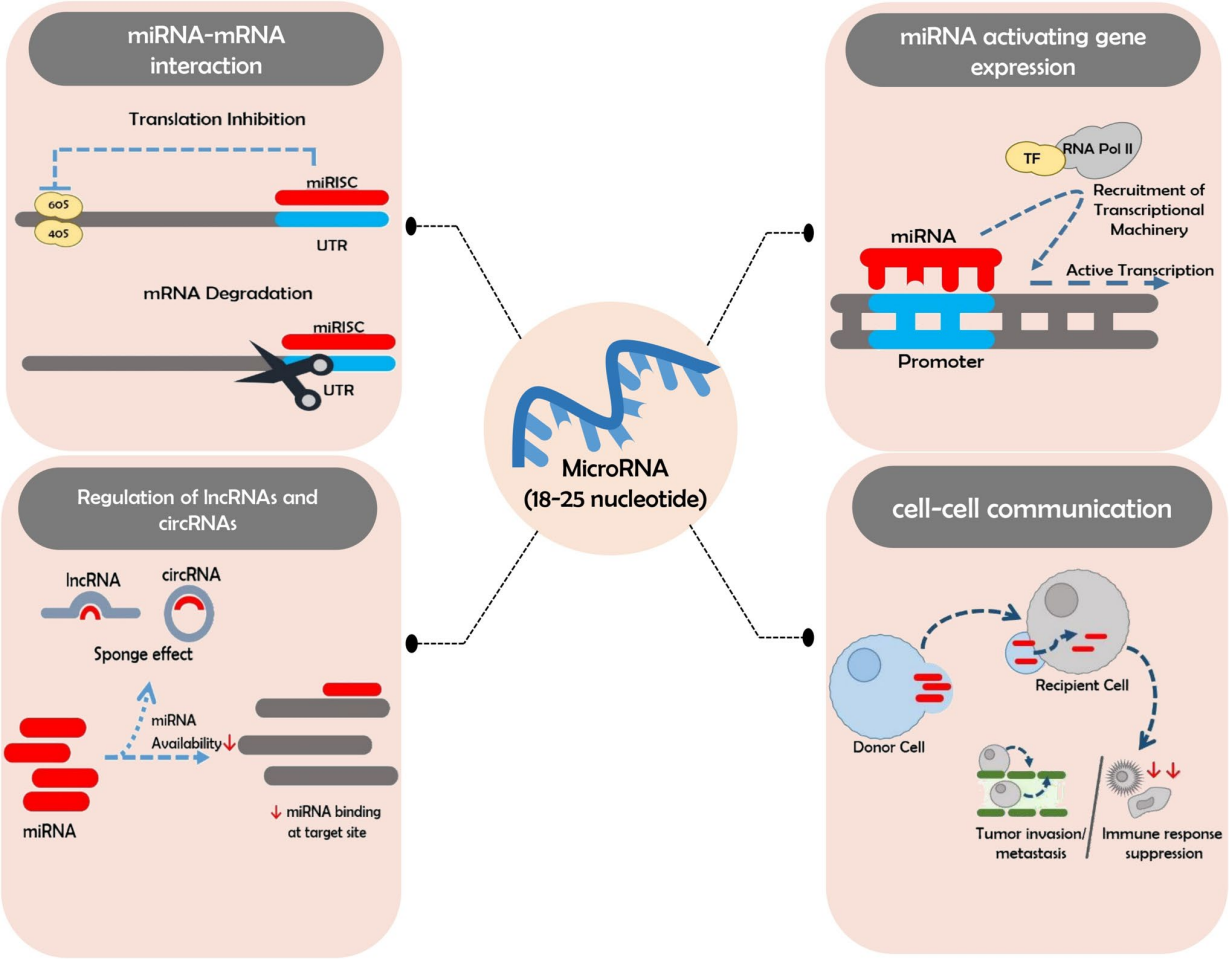
Functional roles of MicroRNAs in gene regulation and cell communication: this figure illustrates the diverse regulatory functions of microRNAs (miRNAs) in cellular processes. (Top left): miRNAs modulate gene expression by interacting with messenger RNAs (mRNAs), either repressing translation by binding to the 3′ untranslated region (UTR) or leading to mRNA degradation via the RNA-induced silencing complex (miRISC). (Top right): In certain contexts, miRNAs can activate gene expression by interacting with promoters or transcriptional machinery, recruiting RNA Polymerase II (RNA Pol II) and transcription factors (TF) to enhance transcription. (Bottom left): miRNAs also regulate long non-coding RNAs (lncRNAs) and circular RNAs (circRNAs), which can act as competitive inhibitors or “sponges,” sequestering miRNAs and reducing their availability for binding to their mRNA targets. (Bottom right): miRNAs are involved in cell–cell communication, packaged into exosomes and secreted by donor cells. Recipient cells take up these exosomes, where the miRNAs influence gene expression, impacting the tumor microenvironment by promoting tumor invasion, metastasis, and immune response suppression

Furthermore, miRNAs facilitate cell–cell communication via extracellular vesicles like exosomes, impacting the tumor microenvironment and immune responses [14]. Their diverse functions position miRNAs as versatile regulators of gene expression and signaling pathways in osteosarcoma and other cancers. Changes in miRNA expression significantly influence tumorigenesis, invasion, metastasis, and angiogenesis, with many alterations observed in malignancies. Figure 1 summarizes the functional roles of microRNAs in gene regulation and cell communication. Notably, Calin et al. highlighted that over 50% of microRNA genes are located in cancer-associated genomic regions or fragile sites [15]. Depending on their roles, miRNAs can upregulate oncogenes by downregulating tumor suppressor genes or act as oncogenes when overexpressed relative to targeted proteins [16].

Given this multifaceted involvement, this review aims to illuminate the diverse roles of microRNAs in regulating OSa progression, with a focus on their clinical applications. We will explore their potential as diagnostic and prognostic markers and their integration into therapeutic strategies for treating OSa. Additionally, we will address open questions regarding certain microRNAs that have shown promise in other cancers but remain underexplored in OSa.

## MicroRNAs as regulators of the cell cycle

MicroRNAs play a pivotal role in the regulation of the cell cycle within osteosarcoma, influencing critical phases that impact tumor growth and proliferation. These small noncoding RNAs function as either oncogenes or tumor suppressors by targeting specific cellular processes, which underscores their potential as therapeutic agents. This discussion explores how various miRNAs interact with the cell cycle in osteosarcoma, highlighting their roles and potential therapeutic applications.

### MicroRNA promote proliferation of OSa cells

Some miRNAs can promote the transition from one cell cycle phase to the next, thereby accelerating cell cycle progression and potentially enhancing tumor growth. MiR-299-5p targets the CDK family, allowing osteosarcoma cell line 63 to progress beyond the G0/G1 phase [17]. MiR-106b is associated with higher disease stages and lung metastasis, taking part in G1/S transitioning; its inhibition reduces cell proliferation and migration [18]. Furthermore, miR-9 enhances osteosarcoma growth by facilitating cell proliferation and progression through the G1 phase of the cell cycle [19]. Similarly, miR-95, by targeting the sodium channel epithelial 1α subunit (SCNN1A), allows cells to progress through the G1 phase and inhibits apoptosis [20].

### MicroRNAs induce cell cycle arrest

Several miRNAs contribute to the cell cycle arrest in osteosarcoma by targeting various regulatory pathways and molecules. MiR-329-3p significantly impacts the cell cycle in osteosarcoma by targeting and reducing the expression of TCF7L1, effectively arresting the cell cycle in the G0/G1 phase. The G1/S transition is blocked by the restoration of miR-329-3p, which inhibits CyclinD1 and c-Myc in the Wnt/β-catenin pathway [21]. Furthermore, miR-302b enhances G1 phase cell cycle arrest through controlling proteins including Akt/pAkt, Bcl-2, and Bim, and reducing the expression of cyclin D1 and CDKs [22]. Similarly, miR-671-5p inhibits key cell cycle regulators such as cyclin D1 and CDC34, effectively halting cell cycle progression [23]. The tumor-suppressive role of miRNA-1236-3p is demonstrated by its ability to reduce cell proliferation and induce cell cycle arrest in HOS and U-2OS cells at the G0/G1 phase through the down-regulation of Krüppel-like factor 8 (KLF8) [24]. Another interesting study by Novello et al. has demonstrated that miR-1 and miR-133b significantly impact the cell cycle by altering the expression of the MET protein, a key receptor tyrosine kinase. Overexpression of these microRNAs in U2-OS cell lines leads to a blockage in the G1 phase of the cell cycle, which results in decreased cell invasiveness and motility [25]. In a similar vein, miR-223 enhances this regulatory effect by targeting Ect2, which prompts the upregulation of cell cycle inhibitors p21 and p27 and the phosphorylation of the retinoblastoma protein, causing the arrest of the cell cycle at the G1 phase [26]. MiR-524-5p is essential in tumorigenesis by targeting and inhibiting Cyclin-Dependent Kinase 6 (CDK6), promoting cell cycle arrest in the G1 phase [27]. Additionally, miR-449c could lead to a substantial decrease in cell proliferation and colony formation, by actively targeting and inhibiting the oncogene c-Myc. Low levels of miR-449c in osteosarcoma, often due to DNA hypermethylation, correspond to increased tumor size and more advanced tumor stages. Therefore, miR-449c may have potential as a target for therapeutic intervention [28]. MiR-145-5p was found to be a tumor suppressor as well. It targets and inhibits E2F transcription factor 3 (E2F3), which is involved in the synthesis of DNA. That leads to decreased cell proliferation and induced G1 phase arrest in osteosarcoma cells [29]. Similarly, cell cycle profile analysis revealed that miR-659-3p inhibited osteosarcoma cells' G1/G0 phase exit, migration, and invasion by down-regulating SRPK1 expression [30]. MiR-3928, targeting ERBB3, IL-6R, and CDK6, leads to an increase in G1 phase cells and a decrease in S-phase cells, suggesting its possible therapeutic potential to fight osteosarcoma [31]. MiR-422a targets BCL2L2 and KRAS, supporting G0/G1 phase cell cycle arrest and inducing apoptosis, potentially pointing to its viability as a therapeutic target [32]. Likewise, miR-154-5p inhibits E2F5, Cyclin E1, and CDK2, contributing to G0/G1 arrest and reduced proliferation in MG63 cells [33]. MiR-1258 serves as a tumor suppressor by targeting AKT3, which leads to reduced cell proliferation and enhanced cell cycle arrest at the G0/G1 phase, thus halting tumor progression [34]. Similarly, miR-199a-3p appears to play a tumor-suppressive role by decreasing cell growth and migration, likely through prompting G1 phase arrest and reducing the S-phase cell population. This effect is thought to be due to the downregulation of mTOR and Stat3 expression [35]. Additionally, miR-494 specifically targets CDK6, being the cause of cell cycle arrest at the crucial G1/S-phase transition, making it a potential diagnostic marker and a therapeutic target [36].

### MicroRNAs that inhibit and promote apoptosis of OSa cells

Some miRNAs could promote apoptosis, leading to reduced tumor growth. MiR-329-3p promotes apoptosis by causing cytoskeletal disintegration and nuclear condensation into apoptotic bodies [37]. Furthermore, miR-302b enhances apoptosis through controlling proteins including Akt/pAkt, Bcl-2, and Bim, and reducing the expression of cyclin D1 and CDKs [22]. In the study by Sun et al., overexpression of miRNA-1236-3p in HOS and U-2OS cells was shown to increase apoptosis, primarily through the down-regulation of Krüppel-like factor 8 (KLF8) [24]. MiR-524-5p induces apoptosis in osteosarcoma cells by targeting and inhibiting Cyclin-Dependent Kinase 6 (CDK6) [27]. Similarly, MiR-449c leads to cell death by targeting and inhibiting c-Myc [28]. MiR-422a induces apoptosis by targeting BCL2L2 and KRAS [32].

### MicroRNA that inhibit and promote OSa cell migration

MiRNAs are found to influence the migration and invasiveness of osteosarcoma cells, affecting metastatic potential. Regarding cell migration, miR-1 and miR-133b reduce cell invasiveness and motility by altering the expression of the MET protein [25]. MiR-659-3p restricts cell migration and invasion by down-regulating SRPK1. Restoring miR-659-3p expression in osteosarcoma cells and in vivo models significantly restricts tumor progression and lung metastasis. However, more research is needed to learn the impact of miR-659-3p on osteosarcoma metastasis [30]. MiR-199a-3p reduces cell migration by downregulating mTOR and Stat3, contributing to G1 phase arrest and limiting the S-phase population [35]. Conversely, miR-9 enhances the metastatic capability of osteosarcoma cells through the regulation of MMP-13 and E-cadherin [19]. MiR-106b promotes cell migration and is linked to advanced disease and metastasis as it facilitates cell cycle progression and thus enhancing movement and proliferation of osteosarcoma cells [18].

**Fig. 2:**
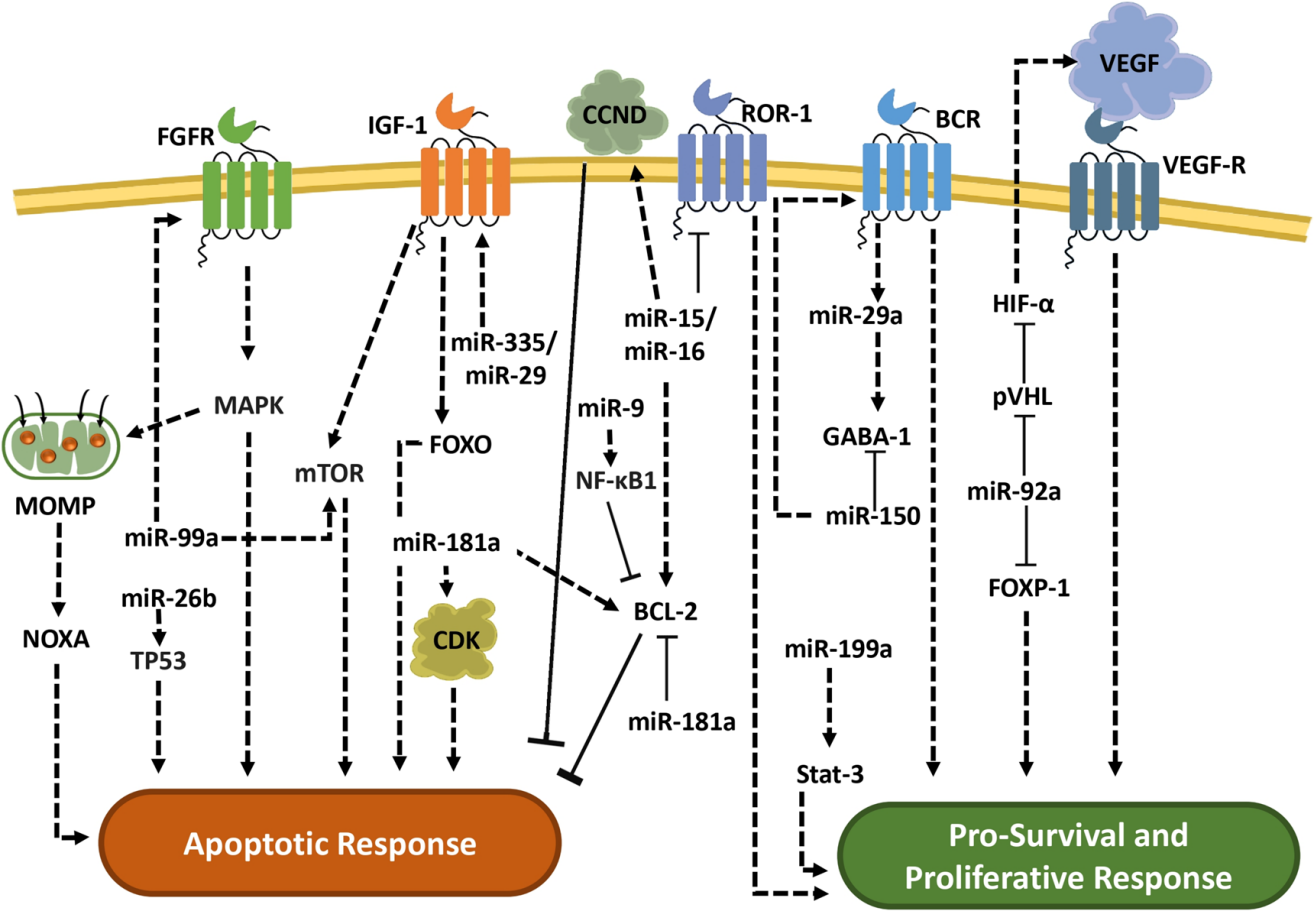
MicroRNA-mediated regulation of apoptotic and pro-survival signaling pathways in osteosarcoma. This schematic illustrates the role of various microRNAs (miRNAs) in modulating key cellular pathways involved in osteosarcoma. Solid lines indicate inhibition, while dashed lines represent regulation

MicroRNAs play a crucial role in osteosarcoma, functioning both as oncogenes and tumor suppressors. Their impact on cell cycle regulation, apoptosis, and cell migration presents significant opportunities for novel therapeutic approaches. Figure 2 schematically summarizes the complex roles of a few microRNAs in regulating apoptotic and proliferative responses. For example, miRNAs such as miR-99a, miR-26b, miR-181a, and miR-29 influence apoptotic processes by targeting key proteins like TP53, NOXA, CDK, and FOXO. Conversely, pro-survival and proliferative signaling pathways are enhanced by miRNAs such as miR-150, miR-92a, miR-199a, and miR-335, which regulate proteins like VEGF, HIF-α, BCL-2, and NF-kB. This dual functionality of miRNAs underscores their pivotal roles in both apoptosis and tumor progression in osteosarcoma. As research advances, targeting specific microRNAs holds the potential to develop more precise treatments that could effectively inhibit tumor growth and metastasis, ultimately improving patient outcomes in osteosarcoma therapy.

## MicroRNA-based approaches in osteosarcoma: diagnostics, prognostics, and treatments

MicroRNAs (miRNAs) are emerging as pivotal tools in the diagnosis and understanding of osteosarcoma. While circulating miRNAs enable minimally invasive diagnosis, with certain miRNAs associated with advanced disease stages and treatment response; tissue-based miRNAs provide critical insights into disease biology, with specific miRNAs correlating with tumor grade and aggressiveness. Moreover, miRNA expression also holds promise as a prognostic marker, as certain miRNAs are linked to improved patient survival. Additionally, miRNAs can regulate genes involved in drug resistance, offering potential strategies to combat OSa. Overall, deciphering miRNA profiles could play a crucial role in diagnosing, predicting prognosis, and guiding treatment decisions for osteosarcoma patients.

### Circulating microRNA as biomarkers of osteosarcoma

Several miRNAs have shown potential as diagnostic markers and are associated with various clinical features of osteosarcoma. Among these, miR-375 stands out due to its significantly lower serum levels in OSa patients compared to healthy individuals [38, 39]. This miRNA is closely linked with advanced stages of the disease, larger tumor size, metastasis, and a poor response to preoperative chemotherapy [40]. Notably, miR-375 can not only distinguish between OSa patients and healthy individuals but also help identify patients with a good pathologic response from those with a poor response [39].

Similarly, miR-22 is a promising diagnostic marker for osteosarcoma (OSa), as it is significantly downregulated in OSa patients compared to healthy controls [41]. In a study by Wang et al. involving 52 patients with surgically resected paired osteosarcoma samples, patients with lower miR-22 expression levels experienced poorer overall survival and disease-free survival [42]. Additionally, miR-22 impedes grade progression in OSa by targeting HMGB1 and inhibiting HMGB1-mediated autophagy, making it an excellent candidate for identifying high-grade tumors [43].

Changes in miRNA levels before and after surgical intervention in osteosarcoma patients provide valuable insights into the disease's progression and response to treatment. Lian et al., involving plasma samples from 50 patients before surgery and 1 month after surgery, along with 90 healthy individuals, observed that the levels of four plasma miRNAs (miR-195-5p, miR-199a-3p, miR-320a, and miR-374a-5p) were significantly higher in OSa patients but significantly decreased following surgery [44]. Similarly studies observed that miR-337-3p, miR-484, miR-582, and miR-3677 were notably lower in both tumor tissues and serum samples from OSa patients compared to healthy individuals, with levels returning to normal post-surgery [45]. While further functional validation is required for these microRNAs they could be used as biological signatures for OSa.

### Tissue-based microRNA as a biomarker of osteosarcoma

Tissue-specific microRNAs are emerging as critical biomarkers for osteosarcoma due to their unique expression patterns within tumors. These patterns provide valuable insights into disease biology, enable correlation with patient prognosis, making them powerful tools for diagnosing and managing osteosarcoma. For example, a study involving 49 patients, comparing osteosarcoma and healthy tissue samples, revealed a significant downregulation of miR-199b-3p in tumors [46]. This is noteworthy because miR-199b-3p is a key regulator of the Wnt/beta-catenin signalling pathway, which plays a major role in osteosarcoma progression [47]. The importance of the miR-199b-3p and Wnt/beta-catenin signalling duo has been highlighted in other cancers, such as colorectal and prostate, where miR-199b-3p inhibits cell proliferation by downregulating cysteine-rich motor neuron 1 (CRIM1) through the Wnt/beta-catenin pathway [48, 49].

Similar studies conducted on a cohort of 15 patients revealed a four-fold increase in miR-4295 expression levels. Furthermore, transfecting osteosarcoma cell lines with miR-4295 mimics and inhibitors showed significant differences in their proliferation abilities [50]. This finding is particularly significant because miR-4295 targets the conserved domain at the 3’UTR of the Interferon Regulatory Factor 1 (IRF1) protein, a known tumor suppressor involved in various cancers [51]. Although the link between IRF1 and osteosarcoma has not been extensively studied, the critical regulatory role of IRF1 in other cancers suggests a promising direction for future research.

A study involving 162 osteosarcoma patients found that miR-34a expression was lower in OSa tissue samples compared to healthy tissues. Interestingly, serum miR-34a levels mirrored those in tissue, and the study also found that these levels normalized after chemotherapy [52]. These findings are significant as they suggest that monitoring tissue-based and circulating miR-34a levels could serve as an alternative technique to assess chemotherapy effectiveness. Given that this approach has already been validated in other cancer types, it could be readily adopted for use in osteosarcoma [53, 54].

### MicroRNA correlation with grade progression in osteosarcoma

Osteosarcoma can be classified based on both its grade and its stage, each of which plays a critical role in determining the aggressiveness and spread of the disease. Tumor grade refers to how abnormal the cancer cells appear under a microscope, with low-grade tumors (Grade I) resembling normal cells and growing more slowly, while high-grade tumors (Grades II and III) have highly abnormal cells and tend to grow and spread rapidly. Most osteosarcomas are high-grade and, as a result, carry a higher risk of metastasis, meaning the cancer can spread to distant organs such as the lungs. In contrast, low-grade osteosarcomas are typically more localized, meaning they remain confined to the bone or nearby tissues and are less likely to metastasize. Therefore, the grade of the tumor is a key factor in determining whether the osteosarcoma is likely to remain localized or progress to a metastatic stage.

MicroRNAs (miRNAs) play a pivotal role in influencing tumor grade and progression in osteosarcoma by regulating critical pathways involved in cell growth, invasion, and metastasis. Their differential expression could serve as prognostic indicators and potential therapeutic targets, offering insights into disease biology and guiding personalised treatment strategies. For instance, in a study involving 48 paired cancerous tissues and adjacent non-tumor tissues, miR-449c exhibited a three-fold decrease in expression levels in tumor samples. Also, miRNA levels decreased two-fold between grade I and grade II/III tumors [28]. The research also showed that miR-449c regulates tumor progression by targeting *c-Myc* which is an upstream target of CDK1, CDK2, CDK4 and CDK6 [55].

Similarly, an investigation with 80 patients revealed higher levels of miR-29 family (miR-29a, miR-29b, and miR-29c) in osteosarcoma patients, with a positive correlation observed between tumor grade and miR-29a and miR-29b expression levels [56]. An identical test carried out on 108 osteosarcoma patients reported expression levels of miR-221 were significantly upregulated in both osteosarcoma tissues and patients' sera. Osteosarcoma patients with distant metastasis and advanced clinical stages exhibit notably higher serum miR-221 levels compared to those without metastasis and in early clinical stages [57]. Another study involving 114 osteosarcoma patients, 40 periostitis patients, and 50 healthy controls found that patients with stage III osteosarcoma had significantly lower levels of miR-124 in their blood compared to those with stage II disease. Similarly, patients with osteosarcoma who had distant metastasis had noticeably lower blood levels of miR-124 compared to patients without metastasis [58]. Though the pathway influenced by these microRNAs is not well understood, this shows the usability of miRNA expressions to validate the tumor grade.

### MicroRNA correlation with overall survival in osteosarcoma

The overall survival of patients is a crucial clinical aspect, particularly in cancer prognosis. While there are no clinical test reports available directly comparing microRNA levels and overall survival over the lifetime of osteosarcoma patients, most studies rely on statistical analyses to elucidate this relationship. These analyses highlight the potential of miRNAs as prognostic markers in understanding patient outcomes. For instance, a study involving 43 osteosarcoma patients revealed that those with elevated serum levels of miR-146a-5p, miR-1260a, miR-487b-3p, miR-1260b, and miR-4758-3p had better survival-rates compared to those with lower levels. Specifically, high serum levels of miR-1260a were associated with significantly higher overall survival, as well as improved metastasis-free and disease-free survival-rates [59]. Additionally, another study with 133 patients demonstrated that miR-95-3p—a key upstream regulator of cyclin D1- was severely downregulated in both low and high-grade osteosarcoma. Patients with higher levels of miR-95-3p experienced notably extended survival compared to those with lower levels [60].

In recent studies, microRNAs associated with the gene locus 14q32 have gained significant attention. Separate studies conducted by Kelly et al. and Sarver et al. confirmed an inverse correlation between overall survival with the expression of 14q32 miRNAs in a series of clinically annotated samples from human osteosarcoma patients. Kelly et al. found that out of 32 deregulated microRNAs in osteosarcoma, 19 belonged to the 14q32 region. Both independently conducted studies produced similar data points, underscoring the significant roles of miR-382, miR-134, and miR-544 in this region. Further functional validation is needed to elucidate the specific pathways these microRNAs affect and their contribution to tumor progression control [61, 62].

A key microRNA in osteosarcoma aetiology is miR-9, which acts as an oncogene promoting tumor proliferation. It is a major regulator of two critical pathways in osteosarcoma: the Wnt signalling pathway and the Hippo signalling pathway [63, 64]. Osteosarcoma patients exhibited a two-fold increase in miR-9 expression [63]. Notably, patients with high miR-9 expression had a 5-year overall survival rate of 16.2%, compared to 60.6% for those with low miR-9 expression, indicating a significant difference in survival-rates correlated to miR-9 levels [65]. While these statistics are compelling, further research is needed to monitor microRNA levels and overall survival over extended periods to validate these findings and provide deeper insights into the pathways involved in osteosarcoma.

### MicroRNA in chemotherapy and drug resistance in osteosarcoma

Chemotherapy with standard agents like methotrexate/doxorubicin/cisplatin also known as the MAP regimen, combined with surgical resection has been the standard procedure for treating localized tumors. OSa is often treated using cisplatin (CDDP) combined with other drugs. Numerous studies have demonstrated that miRNAs can directly or indirectly target the mRNA of specific genes and regulate CDDP drug resistance. Apurinic and Pyrimidine Endonuclease 1 (APE1), which is typically overexpressed in osteosarcoma and positively correlated with angiogenesis, can be targeted and inhibited by miR-765, thereby reducing DNA repair capability and enhancing sensitivity to CDDP [66, 67]. In addition to miR-765, APE1 has binding sites for miR-99b, miR-31, and miR-652. These microRNAs have been shown to regulate cisplatin resistance in other cancers, but their effectiveness in osteosarcoma has not yet been tested, presenting an opportunity for further exploration [68–70].

A major challenge in cancer therapy is the frequent development of drug resistance, which allows cancer cells to proliferate rapidly and become more aggressive, increasing the likelihood of metastasis to other organs. The issue is exacerbated when cancer cells exhibit resistance to multiple drugs, known as Multiple Drug Resistance (MDR). Certain miRNAs, such as miR-375 and miR-138, are significantly elevated in osteosarcoma cells and contribute to their adaptation to chemotherapy [40, 71]. Conversely, reduced expression of miRNAs like miR-199 and miR-21 is associated with the upregulation of MDR proteins. These miRNAs typically regulate the expression of MDR-related proteins, so their downregulation leads to chemoresistance [72, 73]. For example, overexpression of miR-506 in osteosarcoma cells enhances oxaliplatin sensitivity by inhibiting MDR1/P-gp expression through the downregulation of the Wnt/β-catenin pathway [74].

### MicroRNA-based treatments

Traditional drugs often struggle to access their targets due to the complex 3D structure of tumors, whereas miRNAs, due to their small size, can readily access these sites. Additionally, miRNAs have a unique ability to influence gene expression not only within their cells of origin but also in neighbouring cells via gap junctions or exosomes [75]. This allows them to function in a more interconnected and dynamic manner in the tumor microenvironment.

One of the remarkable features of miRNAs is their ability to target multiple mRNAs simultaneously, while conversely, a single mRNA can be regulated by several miRNAs. Each miRNA is estimated to recognize 100–200 target sites across the transcriptome, and approximately 1000 copies of a miRNA per cell are typically sufficient to inhibit its targets effectively [75, 76]. MiRNA binding sites are not limited to the 3'UTR of mRNAs but extend to the 5'UTR, coding regions, and even promoter regions. This broad targeting capability, coupled with the context-specific variations in cell type and state, grants miRNAs immense potential in regulating gene expression and makes them attractive therapeutic targets.

In addition to the direct roles of miRNAs in cancer, emerging studies have also revealed a critical interplay between circular RNAs (circRNAs) and miRNAs in osteosarcoma, further complicating the regulatory landscape. For instance, circ_0000376 has been shown to be upregulated in osteosarcoma, where it promotes cell growth, invasion, and glycolysis by sponging miR-577. This action reduces miR-577's ability to target key metabolic enzymes such as HK2 and LDHA, ultimately facilitating osteosarcoma progression [77]. Similarly, exosome-delivered miR-486-3p has demonstrated the ability to inhibit osteosarcoma progression by targeting the circKEAP1/MARCH1 axis [78]. These findings underscore the complex regulatory network where circRNAs modulate miRNA activity, influencing gene expression and contributing to the pathophysiology of osteosarcoma.

Given this versatility, miRNA-based therapies have garnered significant attention in clinical trials for various human diseases, including cancer. Based on their altered expression patterns in pathological conditions, miRNA therapies can be broadly divided into miRNA mimics and antagonists (antagomirs) [79–82]. MiRNA mimics aim to restore the function of miRNAs that are downregulated in diseases, while antagomirs are designed to inhibit overexpressed miRNAs [83–86]. By regulating specific miRNA alterations, these therapeutic approaches can normalize gene regulatory networks and signaling pathways, effectively reversing cancerous phenotypes. Although these techniques have not yet been implemented in cancer treatments, they hold great promise as less invasive alternatives to traditional therapies.

Although research into the miRNA network specific to osteosarcoma is still in its early stages, the potential for miRNAs to serve as both biomarkers and potent therapeutic agents is highly promising. Continued exploration of these therapies, alongside our growing understanding of circRNA-miRNA interactions, may pave the way for novel, targeted treatments that address the underlying genetic and molecular mechanisms driving the disease. Table 1 summarises a list of important microRNAs along with their function and clinical applications.

**Table 1:** Key microRNAs and their functional roles in cancer progression and clinical applications

MicroRNA	Function	Clinical application	Reference
miR-22	Inhibits HMGB1-mediated autophagy	Biomarker; Grade progression	[42, 43]
Mir-199-3p	Inhibits cell proliferation by downregulating cysteine-rich motor neuron 1 (CRIM1) through the Wnt/beta-catenin pathway	Biomarker	[46, 47]
Mir-4295	Targets the conserved domain at the 3’UTR of the Interferon Regulatory Factor 1 (IRF1) protein	Biomarker	[50, 51]
Mir-449c	Regulates tumor progression by targeting *c-Myc*	Grade Progression	[28]
miR-29a	Lysine demethylation 5B (KDM5B). KDM5B could specifically reduce the methylation levels of histone H3 at lysine 4 (H3K4)	Grade Progression	[56]
miR-95-3p	miR‑95 promotes osteosarcoma growth by targeting SCNN1A and cyclin D1	Biomarker, Overall Survival	[59, 60]
miR-382	suppresses the progression of cancer through the PI3K/Akt signaling pathway by inhibition of Anxa3	Therapeutics, Overall Survival	[61, 62]
miR-765	Supersses Apurinic and Pyrimidine Endonuclease 1 (APE1) thereby reducing DNA repair capability and enhancing sensitivity to CDDP	Enhancement of drug efficiency	[66, 67]
miR-506	Enhances oxaliplatin sensitivity by inhibiting MDR1/P-gp expression through the downregulation of the Wnt/β-catenin pathway	Enhancement of drug efficiency	[74]

## Future perspectives and conclusion

MiRNA therapeutics in cancer present a promising strategy that addresses many limitations of conventional pharmaceutical medications. As endogenous natural molecules, miRNAs offer significant advantages over small-molecule drugs, which typically target single proteins. miRNAs can modulate multiple genes within a regulatory network, enhancing their therapeutic potential compared to synthetic biomolecules. Unlike traditional drugs, which often fail to target "non-druggable" proteins, miRNAs can regulate a variety of pathways and inhibit targets considered otherwise unreachable. This ability broadens their scope in cancer therapy and allows for the rapid identification and optimization of target compounds.

In osteosarcoma treatment, miRNAs show considerable promise in overcoming drug resistance. By modulating key pathways involved in tumor growth and survival, miRNAs have the potential to enhance chemotherapy efficacy. Additionally, emerging miRNA-based therapies, including nanoparticle-mediated delivery systems and exosome-based treatments, highlight their innovative and therapeutic scope. Investigating the regulatory roles of miRNAs thus offers a potential avenue for developing more effective cancer treatments.

However, the application of miRNA therapeutics in osteosarcoma is not without significant challenges. Ensuring the efficient and targeted delivery of miRNAs to osteosarcoma cells remains a major obstacle due to the inherent instability of miRNAs. Off-target effects pose another concern, as miRNAs can interact with multiple mRNAs, leading to unintended gene regulation and potential toxicity. Tumor heterogeneity, both genetic and epigenetic, further complicates therapy design, while the tumor microenvironment can influence miRNA activity. Long-term studies are essential to assess the safety and non-toxicity of miRNA-based treatments, and regulatory approval involves extensive testing, increasing both time and cost for development.

Moreover, miRNA therapies must address the challenge of potential tumor resistance, necessitating an understanding of the mechanisms behind resistance and strategies to overcome them. The high production costs and difficulties in scaling distribution limit the accessibility of miRNA therapies. Additionally, robust clinical trials are essential for establishing the therapeutic benefits and risks of these therapies. Identifying suitable patient populations is crucial for ensuring clinical success.

Despite these challenges, the potential of miRNA therapeutics in osteosarcoma is vast. Their ability to modulate multiple targets and pathways, particularly those involved in drug resistance, presents a novel and promising path forward. As research progresses, overcoming delivery and safety issues will be critical to fully realizing the potential of miRNAs. Continued collaboration among researchers, clinicians, and regulatory bodies is essential to the development of safe, effective, and accessible miRNA-based treatments for osteosarcoma.

## Data Availability

The authors declare that all data and materials supporting the findings of this study are available in this article. Data supporting the findings of this study are available from the corresponding author upon reasonable request.
